# The Clinical Utility of Droplet Digital Polymerase Chain Reaction (PCR): A Breakthrough for Cancer and Noninvasive Prenatal Genetic Disease Diagnosis

**DOI:** 10.7759/cureus.105766

**Published:** 2026-03-24

**Authors:** Shafinaz Khan, Md. Fazlul Kabir

**Affiliations:** 1 Clinical and Diagnostic Services, icddr,b, Dhaka, BGD

**Keywords:** cell free fetal dna, circulating tumor cell, droplet digital polymerase chain reaction, non-invasive prenatal disease, polymerase chain reaction

## Abstract

Recently, oncology practice has undergone a paradigm shift, emphasizing precision-driven and patient-centered treatment models. Droplet digital polymerase chain reaction (ddPCR) is considered a top-tier technique for detecting cancer-associated genetic changes due to its high precision and reliability. Today, ddPCR is widely used for absolute quantification of alleles, identification of rare mutations, assessment of copy number variations, evaluation of DNA methylation, and detection of gene rearrangements in diverse clinical samples. It has proven especially valuable in the analysis of archival tumor tissues, where degraded DNA and limited material often hinder traditional methods, offering objective and automated quantification even under challenging conditions. Additionally, new avenues are emerging that leverage other body fluids such as cerebrospinal fluid and urine, further expanding the potential of ddPCR-based molecular testing. It can also be considered a tool for noninvasive prenatal diagnosis and neonatal screening. This article provides a comprehensive overview of the current and evolving applications of ddPCR in oncologic molecular screening and pediatric-onset genetic disease research.

## Introduction and background

Currently, tumor genotyping has gained importance in clinical oncology, and it is becoming increasingly clear that human malignancies are highly heterogeneous, with their origins and progression tied to a complex spectrum of genetic alterations [1]. These molecular features, in turn, are critical determinants of the therapeutic response and the emergence of resistance to targeted agents. Particularly problematic for standard analytical approaches are mutations that occur at very low frequencies at diagnosis or that evolve during treatment as resistance-conferring alterations [2]. Moreover, many genetic biomarkers, which are often challenging to detect, have been introduced for monitoring disease, enabling ongoing surveillance and early recognition of tumor progression. Across these clinical contexts, detection approaches must be extremely sensitive and highly specific [3]. Droplet digital PCR (ddPCR) is now recognized as a pivotal tool in cancer research and clinical applications owing to its superior sensitivity and precision relative to traditional analytical methods [4-8]. 

In dPCR, a single PCR is split into thousands of tiny partitions, enabling the physical separation of individual template molecules before amplification. Partitioning can be achieved through multiple methods, such as oil-suspended microdroplets (ddPCRs), engineered microwells, or microfluidic valving technologies [6]. After endpoint amplification in many separate partitions, fluorescence is measured to mark each unit as amplified or not amplified. These counts are then applied to Poisson calculations to infer the original target copy number [9].

This article is a narrative review, and the literature was selected based on relevance and significance to the topic rather than a systematic search strategy. In this review, we highlight the key clinical and research applications of ddPCR in oncology and liquid biopsy, paying particular attention to alternative biofluids that are not yet routinely used but are becoming increasingly relevant in different cancers. We also consider how this technology may be applied to noninvasive prenatal testing for diseases such as muscular dystrophy and other genetic anomalies.

## Review

Applications of ddPCR for molecular analysis of FFPE (formalin-fixed paraffin-embedded) tissue samples

This diagnostic approach has emerged as a promising substitute for conventional techniques used in routine clinical settings to measure molecular markers in FFPE tissue. A common challenge with FFPE-derived DNA is its degradation, which significantly hinders the performance of many molecular assays. Because ddPCR targets very small DNA fragments, it can measure genetic changes in compromised samples with substantial background normal DNA sequences [10]. This was illustrated in evaluating fibroblast growth factor receptor 2 (FGFR2) amplifications in FFPE gastrointestinal adenocarcinomas, where ddPCR showed greater sensitivity than traditional real-time PCR [5]. ddPCR has also proven valuable for detecting loss of heterozygosity (LOH) in the BRCA2 gene, an important molecular event in breast cancer, which is typically difficult to assess accurately via standard analytical approaches [10].

Multiple studies have evaluated human epidermal growth factor 2 (HER2) status in FFPE samples from breast and gastric cancers via ddPCR, reporting results consistent with those obtained via fluorescence in situ hybridization (FISH) and immunohistochemistry (IHC), the standard reference methods [11-16]. Across these investigations, ddPCR reliably quantified the HER2 copy number at both the DNA and RNA levels [11,17]. Other studies reported that ddPCR can identify Kristen rat sarcoma viral oncogene (KRAS) mutations from very limited quantities of DNA extracted from FFPE samples in non-small cell lung cancer [18] and multiple gene fusions, anaplastic lymphoma kinase (ALK), ROS proto-oncogene 1 (ROS1), and RET (REarranged during Transfection) associated with bronchogenic carcinoma at the same time [19].

Role in targeted therapy

Precision therapies focus on tumor-specific molecular pathways, but gene mutations remain a key mechanism behind resistance to these agents [20]. In non-small cell lung cancer, elevated epidermal growth factor receptor (EGFR) activity is frequently observed [21], making EGFR tyrosine kinase activity an established treatment option, whereas acquisition of the EGFR T790M variant is linked with the diminished response to drugs [22]. Zhang et al. reported that ddPCR was able to detect EGFR mutations [23]. These results highlight ddPCR as a valuable approach for the early identification of resistance that develops during tyrosine kinase inhibitor (TKI) treatment. Furthermore, it has gained regulatory authorization for clinical studies assessing plasma EGFR mutation to track therapeutic effectiveness and forecast resistance in late-stage patients [24]. Another study reported that ddPCR may serve as a viable noninvasive strategy for tracking TKI therapy by identifying EGFR mutations in urine [25].

Evaluation of cancer advancement and therapeutic effectiveness

Cancer-associated genetic alterations undergo continual changes during disease progression following therapeutic spread and potential recurrence. Olmedillas et al. [26] observed that individuals with colorectal carcinoma showed higher circulating levels of both wild-type KRAS and the KRAS G12V mutant allele. In 2016, Takeshita et al. [27] showed that in patients with estrogen-positive breast cancer, the proportion of Estrogen Receptor 1 (ESR1) mutations detected in circulating cell-free DNA varied over the course of therapy, and higher mutation prevalence was linked to reduced therapeutic benefits. Applications of ddPCR are well established in hematologic cancer research. In 2016, Minervini et al. [28] documented a more frequent occurrence of Neurogenic locus notch homolog protein 1 (NOTCH1) mutations in chronic lymphocytic leukemia patients using this technique.

Minimal residual disease (MRD) is characterized by a low burden of residual cancer cells in patients undergoing or following therapy, a term frequently applied in the management of hematological cancers. Cancer relapse is most commonly attributed to the persistence of MRD [29]. Emerging studies indicate that ddPCR holds significant promise for the assessment of minimal residual disease. Guerrini et al. [30] reported that ddPCR can be used to track MRD and determine the therapeutic progress in patients with hairy cell leukemia, as it outperformed qPCR in identifying serine/threonine protein kinase B-raf (BRAF) mutations, with a lower detection threshold (0.005% vs. 0.025%). Moreover, Luisa et al. [31] established that the exact amount of calreticulin gene mutations can be accurately measured by using this technique.

Clinical and research applications in liquid biopsy

Liquid biopsy has gained prominence as a powerful technique, offering a complementary or conditional alternative to traditional tissue biopsy for tumor genotyping [32]. Its noninvasive nature, ease of collection, and suitability for longitudinal sampling are notable advantages [33]. This method focuses on cell-free DNA in the bloodstream, particularly tumor-derived DNA, and checks for tumor cells that are circulated in the blood [3]. Several studies suggest that serial cfDNA testing may be more informative clinically than monitoring based on circulating tumor cells [34].

Notably, patients whose serum ctDNA levels became undetectable after initiating treatment experienced improved clinical outcomes and survival, and rising ctDNA levels predict recurrence months before it is detectable by computed tomography (CT) scans [35]. Some studies have reported that PIK3CA mutations and HER2 amplification have been found with more frequency in malignancies related to the pancreatobiliary system [36] and the GIT [13], respectively, and support the use of serum as a practical cell-free DNA source. Similarly, Combaret et al. [37] reported that both plasma and serum could be used interchangeably to detect ALK hotspot mutations, indicating that ctDNA suitable for analysis can be obtained from either fluid.

Among cancers analyzed via cfDNA, lung cancer represents a major field in which ddPCR has shown significant clinical relevance. The reported analytical sensitivities for EGFR mutation detection in plasma range from 0.005% to 0.01%, which is equivalent to detecting 5-50 mutant copies within 10000 wild-type copies [27], with similar detection thresholds described for T790M [38]. Some studies have noted slightly higher limits of detection, such as 0.04% [39-41]. In addition to analytical sensitivity, clinical sensitivity is frequently reported, defined by Lee et al. [23] as the proportion of tissue-positive cases that are also plasma positive, whereas specificity reflects the proportion of plasma- and tissue-positive cases that are also plasma positive, whereas specificity reflects the proportion of plasma- and tissue-negative samples among all negative tissue cases. Published data indicate sensitivities of 61-82% and specificities between 63% and 100% [23,39,42-46]. Notably, ddPCR has been proven effective in revealing tumor heterogenicity, a characteristic feature of NSCLC, as evidenced by the detection of dual EGFR mutations in patients whose tumor biopsies indicated only a single mutation [47]. Comparable findings of intratumoral heterogeneity using ctDNA have been reported in neuroblastoma patients [37].

Although ddPCR-based cfDNA analyses predominantly emphasize rare variants, such as the EGFR T790M mutation, this platform is equally suited for evaluating broader classes of genetic alterations, including structural rearrangements. In metastatic breast cancer, ddPCR has successfully identified HER2 amplification [48], and quantification of these rearrangement-derived fragments can reveal a signal of relapse or progression months before they become apparent through standard imaging techniques [49].

Circulating Tumor Cells

Circulating tumor cells are increasingly recognized as either substitutes for or complements to cfDNA as genetic analytes for tumor profiling. ddPCR has been successfully used as a platform to verify genetic alterations found through targeted deep sequencing of CTC from individuals associated with bronchogenic carcinoma [50]. Another study reported that ddPCR can be used to identify BRAF mutations in CTCs from metastatic melanoma patients when combined with whole-genome amplification [51]. In advanced CRPC, ddPCR can detect AR-V7 in as few as five CTCs [52].

Analysis of miRNA expression

In cancer detection, microRNAs have emerged as promising circulating biomarkers for the early diagnosis of malignancies [24,53-54]. Digital PCR, particularly dPCR, facilitates the absolute quantification of specific microRNAs in lung tissue samples [55]. Researchers have used a microwell-based dPCR platform to identify the copy numbers of five microRNAs linked to bronchogenic carcinoma [56]. Another study by Li et al. applied dPCR to measure two bronchogenic carcinoma-related microRNA levels (miR-31, miR-210) in clinical specimens [57].

Clinical relevance of analyzing various body fluids via ddPCR

Most ddPCR applications in oncology, especially in liquid biopsies, utilize plasma or serum as the primary source of nucleic acids. However, other body fluids can also supply adequate amounts of DNA and RNA for analysis. The performance characteristics of ddPCR in different types of biological fluids are summarized in Table 1.

**Table 1:** Evaluation of diagnostic performance of ddPCR found in different studies ND: Not Done, CSF: Cerebrospinal fluid, EGFR: Estimated glomerular filtration rate

Sample Type	Biomarker	Performance characteristics of ddPCR	Other method used	Reference
CSF	EGFR mutation in Lung adenocarcinoma (CNS metastasis)	Sensitivity:56% Specificity: 89%	ND	[63]
	EGFR mutation in Non-small cell lung cancer (suspected leptomeningeal metastases)	Sensitivity:94% Specificity: 100%	Cytology Sensitivity:76%	[64]
Urine	Prostatic cancer associated DNA methylation biomarker	Sensitivity:100% Specificity: 50%	PSA Sensitivity:100% Specificity: 27.3%	[67]
	Exosome gene panel in prostatic carcinoma	PCA3 Sensitivity:97.2% Specificity: 37.3% PCGEM1 Sensitivity:80.5% Specificity: 38.8%	ND	[68]
	TERT promoter mutation in bladder tumor	Frequency: 92.2 %	ND	[70]
	Y373C mutation in bladder tumor	Frequency: 20.3%	Sanger sequencing Frequency: 30.8%	[71]
	TERT promoter mutation and FGFR2 mutation in urothelial carcinoma	TERT promoter Sensitivity:47.4% Specificity: 100% FGFR2 Sensitivity:16.1% Specificity: 37.3%	Cytology Sensitivity:44.6% Specificity: 96%	[72]
Stool	ITGA6 in colorectal cancer	Sensitivity:78% Specificity: 93%	qPCR Sensitivity:83% Specificity: 93%	[78]
	Fusobacterium nucleatum in colorectal cancer	Sensitivity:54% Specificity: 90%	ND	[81]
	16S gene of H. pylori	Sensitivity:84% Specificity: 100%	Serology Sensitivity:100% Specificity: 71%	[82]
Vitreous fluid	MYD88 L265P gene in Vitreoretinal lymphoma	Sensitivity:75% Specificity: 100%	ND	[85]
Sputum	Methylated gene in Lung cancer	Sensitivity:86.6% Specificity: 90.6%	Cytology Sensitivity:88.8% Specificity: 70.6%	[90]
	Ex19 mutation in lung adenocarcinoma	In cytology positive case: Sensitivity:80% Specificity: 100% In cytology negative case: Sensitivity:3.1% Specificity: 100%	ND	[91]
Bronchoalveolar lavage	miRNA in lung adenocarcinoma	Sensitivity:94.83% Specificity: 77.59%	Cytology Sensitivity:77.59% Specificity: 58.62%	[93]
Pleural fluid	EGFR mutation in non-small cell lung cancer	Detection rate: 75.4%	Sequencing Detection rate: 43.8%	[95]

Cerebrospinal Fluid

Liquid biopsy via ddPCR has gained considerable momentum in recent years as an effective tool for identifying molecular markers in childhood brain malignancy with an emphasis on diffuse midline glioma (DMG) [58,59]. Early investigations used ddPCR to detect any alteration found in the IDH1 gene in CSF, and subsequent studies have expanded its application, particularly in lower-grade gliomas [60]. Current research further supports the usefulness of ddPCR for tracking the disease progress in children developing medulloblastoma. Notably, the integration of NGS with ddPCR facilitated the identification of diverse tumor-associated mutations in CSF, reinforcing its advantage over plasma as a ctDNA source and its superior sensitivity relative to conventional cytology [61]. In addition to primary CNS tumors, CSF-based detection of genetic alterations has also been applied to metastatic CNS disease, including leptomeningeal and brain metastases from lung adenocarcinoma [62-64], breast cancer [65], and melanoma [66].

Urine

Several studies have demonstrated that ddPCR has been found to work well for testing cancer-related markers in urine from patients having urological malignancies. Strategies to enhance the noninvasive diagnosis of high-grade prostate cancer include assessing genetic targets (GSTP1, APC, RASSF1A, PITX2, and C1orf114) in filtered urinary cellular material [67] and developing a two-gene urinary exosomal mRNA panel (PCA3, PCGEM1) [68]. The urinary transcriptome has also emerged as a promising biomarker source and has been recently validated via ddPCR, which has revealed that quantitative analysis of five microRNA targets (FTH1, BRPF1, OSBP, PHC3, and UACA) enabled discrimination among urological malignancy, benign hyperplasia of the prostate, and control groups using as little as 1 ml regardless of centrifugation status [69]. In parallel, ddPCR-based detection of recurrent TERT promoter and FGFR3 in urine is also helpful for initial diagnosis of bladder and upper urinary carcinoma [70-75]. Genetic alterations related to the development of carcinoma, such as FGFR3 and PIK3CA, have also been quantified in plasma and urine from individuals associated with urological malignancy to support disease tracking and treatment assessment, supporting early recognition of metastatic progression [76,77].

Stool

Herring et al. [78] were among the first to demonstrate the feasibility of the use of ITGA6 and ITGA6A transcripts in fecal specimens from individuals with colorectal cancer via ddPCR. Subsequent work analyzing stool-derived DNA revealed the KRAS G12D hotspot mutation in 80% of CRC patients known to harbor this mutation in their tumor tissue [79]. More recent ddPCR studies have detected hypermethylation at GRIA4- and VIPR2-associated CpG islands in CRC stool samples, emphasizing their potential utility as early, noninvasive biomarkers [80]. In addition to genetic and epigenetic tumor markers, ddPCR has been employed to detect bacterial species implicated in malignancy, including *Fusobacterium sp.* in colorectal carcinoma [81] and *H. pylori* in malignancy related to the GIT [82,83]. In a related application, ddPCR-based 16S rRNA profiling revealed altered gut microbiota in postoperative stool samples from patients who underwent pancreaticoduodenectomy, with cancer diagnoses confirmed in 45 of 50 cases [84].

Ocular Fluids

Hiemcke-Jiwa et al. [85] demonstrated that ddPCR-based identification of the MYD88 L265P variant in ocular fluid samples is a practical and dependable approach for both diagnosing vitreoretinal lymphoma and assessing therapeutic progress. In a more recent study, the same mutation was detected in vitreous fluid by using this approach in approximately 75% of VRL patients and in the case of lymphoplasmacytoid lymphoma [86].

Saliva

Saliva has also been evaluated as a source for detecting EGFR mutations in NSCLC patients via ddPCR. Paired plasma and saliva samples demonstrated 83.78% concordance, which aligned with the patient's therapeutic outcome. In contrast, cfDNA concentration measured in saliva was not effective in differentiating patients with NSCLC from healthy participants and individuals with nonmalignant lung conditions, indicating that saliva serves as a qualitative rather than quantitative marker and is therefore unsuitable for diagnostic use [87]. Another comparative investigation demonstrated that the emerging electric field-induced release and measurement (EFIRM) reached complete sensitivity for EGFR mutation detection in both plasma and saliva samples from NSCLC patients [88]. As noted earlier, a PIK3CA variant could be identified in this specimen type from individuals with the PIK3CA-related overgrowth spectrum [89].

Sputum

In 2018, one investigator pioneered the use of droplet digital methylation-specific PCR (ddMSP) to detect sputum-based epigenetic biomarkers and construct a classifier for early lung cancer detection [90]. Subsequent studies have reported the identification of EGFR mutations in sputum samples from NSCLC patients via ddPCR [91,92]. Moreover, Hackner et al. [93] reported that combined detection of EGFR variants in sputum had been shown to increase the diagnostic sensitivity of T790M in patients with progressive disease relative to that of regular specimen plasma testing alone.

Bronchoalveolar Lavage

Bronchoalveolar lavage (BAL), specifically the cellular fraction derived from it, represents another oncologic specimen type analyzed by ddPCR. In 2017, a ddPCR-based approach was applied to verify two miRNA-based models (miR-205-5p and miR-944) for enabling subtype differentiation of NSCLC in bronchoalveolar lavage specimens, demonstrating superior diagnostic performance compared with cytology [93]. Additionally, the assessment of the BRAF V600E mutation in BAL fluid via ddPCR has been suggested as a supplementary diagnostic method for pulmonary Langerhans cell histiocytosis [94].

Pleural Effusion

A number of studies have documented the identification of EGFR variants in pleural effusions from individuals with NSCLC [95-98]. Mutation analysis has been conducted on cfDNA isolated from the supernatant fraction [97], from the cellular pellet [98], or from both [97,98]. Hummelink et al. [99] also reported that genetic alterations in the KRAS and EGFR genes could be identified in this specimen from a variety of cancers, including NSCLC, colon and appendiceal carcinomas, and adenocarcinoma of unknown primary origin.

Pancreatic Juice

ddPCR has been used to assess KRAS mutations in this type of specimen, like bile, in individuals with pancreatic ductal adenocarcinoma and cholangiocarcinoma, and its performance is comparable to that of NGS. These findings suggest that ddPCR-based bile liquid biopsy could be a dependable diagnostic option for pancreatobiliary cancers [98]. In another context, ddPCR has additionally been used for microbial detection in biliary and intestinal contents obtained during a pancreaticoduodenectomy procedure [84].

Applications in noninvasive diagnosis of prenatal genetic disease

The principle underlying noninvasive prenatal diagnosis (NIPD) relies on the ability to detect fetal DNA circulating in maternal plasma [99]. The total amount of cell-free DNA (cfDNA) in peripheral blood is limited and highly variable among individuals, typically corresponding to 600-1000 genome equivalents (GE) per milliliter of plasma [100]. Only a small fraction-approximately 5-15%-of this cfDNA originates from the fetus (cfDNA), and this proportion also varies widely. Consequently, highly sensitive and robust analytical technologies are needed to address these challenges and to accurately detect and quantify the fetal DNA fraction amidst a large background of maternal DNA [99].

Digital PCR has shown strong applicability in this setting. Different types of chromosomal abnormalities can be readily quantified via dPCR [101,102]. Furthermore, droplet digital PCR has demonstrated the ability to detect isochromosome 12p in patients with Pallister‒Killian syndrome [103]. dPCR can also quantify deletions within the DFNB1 locus in cases of deafness [104]. Moreover, dPCR is capable of identifying single-gene deletions associated with disorders such as spinal muscular atrophy type 1 (SMN1) [105,106] and Southeast Asian-type α-thalassemia involving HBA1 and HBA2 [107]. In congenital hemangiomas, rare vascular tumors arising prenatally from somatic mutations, dPCR has enabled the detection of subclonal mutations in GNAQ and GNA11.

In the context of type 1 diabetes, pancreatic β-cell loss is thought to precede the development of hyperglycemia. Thus, early detection of β-cell death would be clinically valuable. Because the promoter sequence of the INS gene is specifically unmethylated in β-cells [108], increased levels of this genetic sequence, detected via dPCR, have been observed in children with recent-onset type 1 diabetes [109].

A landmark demonstration of dPCR for prenatal testing came in 2007, when Dennis Lo and colleagues used digital PCR to detect trisomy 21 in maternal plasma [106]. In a heterozygous individual, the ratio of the two alleles of a gene is normally 1:1 in maternal blood; in homozygous individuals, only one allele is present. In trisomy 21, the fetus carries an extra copy of chromosome 21, leading to triplicate allelic representation of the gene. Two alleles will be identical in heterozygous cases, and one will differ, producing a detectable shift to a 2:1m250115745 ratio in maternal plasma. By measuring this ratio, Lo and his team demonstrated that dPCR could noninvasively diagnose trisomy 21 and other fetal chromosomal aneuploidies, thereby establishing the feasibility of applying digital PCR for prenatal genetic testing.

Challenges and future perspectives

dPCR also has limitations that must be considered. The scope of variant analysis is limited by sample availability. To overcome this restriction, multiplexing approaches have been introduced, such as advanced dPCR systems equipped with expanded optical channels [110]. Moreover, multiple targets can be detected within a single fluorescence channel by adjusting probe concentrations for identically labeled fluorophores or by designing DNA fragments of distinct length combined with intercalating DNA dyes [111]. A further key challenge involves spurious positive signals, which frequently stem from amplification artifacts when low-frequency variants are analyzed against a large excess of normal DNA, thereby restricting the lowest reliably measurable allele fraction [112]. Although infrequent, both false-negative and false-positive results remain possible in ddPCR assays, raising concerns about relying solely on this technique for clinical decision-making. Therefore, experimental artifacts still represent an important area in need of improvement [112].

## Conclusions

Research on ddPCR in oncology is rapidly accelerating, with a marked surge in publications over the past year. Because of its strong analytical performance, ddPCR has emerged as a highly effective technology for cancer research. ddPCR-based molecular markers provide clinically relevant information for assessing therapeutic response and for dissecting the molecular underpinnings of drug resistance across numerous malignancies. However, limitations remain, particularly related to assay scalability, reduced analytical sensitivity in samples with low DNA yield or from tumors that shed minimal DNA, usually early-stage or anatomically isolated, and the current lack of standardized methodologies. Its high precision and sensitivity also support the potential extension of ddPCR to noninvasive prenatal testing for a wider array of monogenic diseases. Further research is warranted to fully establish its utility in these domains.
